# Willingness to administer mouth-to-mouth ventilation in a first response program in rural Bangladesh

**DOI:** 10.1186/s12914-015-0057-8

**Published:** 2015-08-01

**Authors:** Tom Stefan Mecrow, Aminur Rahman, Saidur Rahman Mashreky, Fazlur Rahman, Nahida Nusrat, Justin Scarr, Michael Linnan

**Affiliations:** International Drowning Research Centre – Bangladesh (IDRC-B) of CIPRB, House – B 162, Road – 23, New DOHS, Mohakhali Dhaka, 1206 Bangladesh; Centre for Injury Prevention Research, Bangladesh (CIPRB), Dhaka, Bangladesh; Royal Life Saving Society Australia (RLSSA), Sydney, Australia; The Alliance for Safe Children (TASC), Atlanta, USA

## Abstract

**Background:**

Timely mouth-to-mouth ventilation is critical to resuscitate drowning victims. While drowning is frequent, there are no lay persons trained in cardio-pulmonary resuscitation (CPR) in rural Bangladesh. As part of a feasibility study to create a first response system in a conservative Islamic village environment, a pilot was undertaken to examine willingness to provide mouth-to-mouth ventilation for drowning resuscitation.

**Methods:**

A questionnaire was administered to 721 participants at the beginning of a village-based CPR training course. Trainees were asked regarding willingness to administer mouth-to-mouth ventilation on a variety of hypothetical victims. Responses were tabulated according to the age, sex and relationship of the trainee to the postulated victim.

**Results:**

Willingness to deliver mouth-to-mouth ventilation was influenced by sex of a potential recipient and relationship to the trainee. Adolescent participants were significantly more willing to perform mouth-to-mouth ventilation on someone of the same sex. Willingness increased for both sexes when the postulated victim was an immediate family member. Willingness was lower with extended family members and lowest with strangers. Adult trainees were more likely to perform mouth-to-mouth ventilation than adolescent trainees in any scenario.

**Conclusion:**

Adults express more willingness to resuscitate a broader range of drowning victims than adolescents. However in rural Bangladesh, adolescents are more likely to be in close proximity to a drowning in progress. Further efforts are needed to increase willingness of adolescents to provide resuscitation to drowning victims. However, despite potential cultural limitations, trained responders appear to be willing to give mouth-to-mouth ventilation to various recipients. Final determination will require evidence on response outcomes which is being collected.

## Background

In 2011 the International Drowning Research Centre – Bangladesh (IDRC-B) began a research programme to establish a village-based first response program in rural Bangadesh. Previous research had identified that the most frequent cause of cardiac arrest in villages in rural Bangladesh was the hypoxic arrest experienced in drowning [1]. Although the highest rates of drowning are in children, adults also drown at high rates, therefore responders need to be able to provide mouth-to-mouth ventilation to victims of all ages (Table 1).

**Table 1:** Drowning rates in Bangladesh

Adult drowning rates	
Age group (yrs)	Drowning rates/100,000
18-24	2.0
25-44	1.1
45-64	6.4
65 & above	4.6
18 -65+	2.4
Child drowning rates	
Age group (yrs)	Drowning rates/100,000
1-4	86.3
5-9	26.2
10-17	2.6
1-17	28.6

Source: Bangladesh Health and Injury Survey, Dhaka: DGHS, ICMH, UNICEF, TASC; 2005

Community-based CPR response programmes are common in high-income countries (HICs) and outcomes-based research has shown they are an effective component of the chain of survival [2, 3].

The European Resuscitation Council provide specific guidelines on the resuscitation of drowning victims which include the provision of mouth-to-mouth ventilations. Therefore, the CPR taught in this first response programme incorporates mouth-to-mouth ventilation and compressions, rather than compression-only CPR [4].

The success of this first response programme largely depends on lay-person responders being able to learn CPR as well as their willingness to deliver it in a timely fashion [5]. Although a programme assessment published in 2014 found that participants in Bangladesh were able to learn CPR for drowning resuscitation and other skills needed for first aid, no studies have previously been conducted in Bangladesh that look at willingness to provide mouth-to-mouth ventilations [6].

A key question regarding drowning response was whether willingness to administer mouth-to-mouth ventilation would be influenced by cultural values and relatively conservative religious beliefs prevalent in rural Bangladesh where approximately 90 % of the population is Muslim [7]. A literature review found two studies that addressed willingness to administer CPR in Islamic countries. In Malaysia a study of medical and dental students showed that respondents were less likely to perform mouth-to-mouth ventilations on a member of the opposite sex, or of another race [8]. In Saudi Arabia university students noted that the gender of the victim would be the most important factor in their willingness to perform mouth-to-mouth ventilation [9].

Willingness of trained responders to administer mouth-to-mouth ventilation is essential to a CPR program targeting drowning. The goal of this pilot study was to determine whether rural Bangladeshi villagers would administer mouth-to-mouth ventilation to a variety of hypothesised victims. Age, sex of the responder and sex of the victim as well as relationship with the victim were examined in relation to willingness to administer mouth-to-mouth ventilation.

## Methods

Raiganj, population 3,097,450, is a landlocked rural sub-district where drowning rates are high. 721 participants were recruited to attend a First Response training course in a union (a union is a smallest administrative unit) of Raiganj between March and August 2011. Approximately 70 % of participants were adolescents aged 10-19, split equally by sex. Adolescent participants had at least 1 year of formal school education. Most adult participants were selected from community business leaders, NGO staff and local government officials, all of whom had completed at least secondary schooling.

Prior to starting the course participants completed a self-administered questionnaire to evaluate knowledge of resuscitation prior to commencing the CPR course and willingness to administer CPR. To evaluate participants willingness to perform mouth-to-mouth ventilation, each was asked: *“Imagine that someone collapses in front of you and stops breathing. To bring them back to life you must put your lips to theirs and blow into their mouth.”* Participants indicated their willingness to perform mouth-to-mouth ventilation on relatives, friends and strangers. Trainers assisted participants who had difficulty due to poor literacy. Discussion was not allowed during questionnaire completion. Participants were told results from the questionnaire would have no effect on passing or failing the course. Informed verbal consent was obtained from the participants prior to collect data. The study was approved by the Centre for Injury Prevention and Research, Bangladesh Ethical Review Committee.

### Data Analysis

SPSS (Version 17) was used for the analysis. The data was categorised into adolescent (aged 10-19) and adult (20+) categories. Pearson’s chi-squared tests were used to examine willingness of male and female participants to perform mouth-to-mouth ventilation on victims of different sexes. Fishers Exact Test was used when the frequency of response for a category was less than five. An odds ratio for willingness was calculated for each hypothesised victim.

## Results

Of 721 participants, 234 (32.5 %) were young adolescents aged 10-14, 250 (34.7 %) were older adolescents aged 15-19, and the remaining 237 (32.9 %) participants were adults aged between 19 and 66 years. Overall, 47.2 % were males. Most participants (96 %) were Muslim, and 4 % were Hindu. Participants came from a range of backgrounds. About three quarters (73.2 %) of adolescents were students (accounting for 95 % of the total student sample). The majority of adult participants were civil servants or worked for non-governmental organizations. Only 5.4 % of participants had received any prior first aid training. It is unknown if this training included CPR instruction (Table 2).

**Table 2:** Occupation of participants by age

Occupation	Adolescent Frequency	Adolescent Percent	Adult Frequency	Adult Percent
Civil servant/NGO	0	0.0	125	52.7
Housewife	0	0.0	30	12.7
Student	354	73.1	17	7.2
Unemployed	49	10.1	8	3.4
Skilled labourer	20	4.1	0	0.0
Business	14	2.9	32	13.5
Agricultural activities	10	2.1	12	5.1
Unskilled labourer	7	1.4	0	0.0
Other	30	6.2	13	5.5
Total	484	100.0	237	100.0

### Willingness to perform ventilation by age and sex of participant

Table 3 shows a significant difference between adolescent (aged 10-19) male and female participants in their willingness to perform mouth-to-mouth ventiltion on various hypothesised vicitms. There was no significant difference between young and older adolescent responses. Males were significantly more likely to be willing to perform ventilation on male victims and females were significantly more likely to be willing to perform ventilation on female victims (P < 0.001 in all circumstances).

**Table 3:** Willingness of adolescents to perform ventilation on hypothesized victims by sex

Identity of victim	Male Respondent		Female Respondent		Chi^2^ P value	Odds Ratio	95 % CI
						Male/Female (* = Female/Male)	
	N	%	N	%			
Mother	176	88.0	268	96.4	<0.001	*3.70	1.69 – 7.69
Father	186	93.5	228	82.9	<0.001	2.95	1.55 – 5.62
Brother	188	93.1	217	79.5	<0.001	3.47	1.87 – 6.43
Sister	169	84.1	265	96.0	<0.001	*4.57	2.22 – 9.09
Grandmother	109	63.4	174	80.6	<0.001	*2.39	1.52 – 3.85
Grandfather	132	80.5	95	46.8	<0.001	4.69	2.92 – 7.54
Aunt	112	55.5	213	76.6	<0.001	*2.63	1.79 – 3.85
Uncle	167	83.1	100	35.8	<0.001	8.79	5.65 – 13.69
Male friend	164	80.8	47	16.9	<0.001	20.76	12.98 – 33.19
Female friend	75	37.5	210	75.3	<0.001	*5.00	3.45 – 7.89
Stranger	101	50.0	47	16.9	<0.001	4.94**	3.25 – 7.49

**Sex of stranger not stated

Male and female adolescent participants were most likely to be willing to perform ventilation on close family members (mother, father, brother and sister). The largest difference between male and female adolescent participants was in willingness to perform ventilation on a friend of the opposite sex. The odds of ventilation administered to a male friend were 20.76 (95 % CI: 12.98 – 33.19) times higher in male participants compared to female participants. Similarly, females were five times more likely than males to administer mouth-to-mouth ventilation to a female friend (OR = 5.00, 95 % CI: 3.45 - 7.49).

The proportion of adolescent participants willing to ventilate a stranger (sex of stranger not stated) was 50 % for males and 16.85 % for females. The difference was significant (P = <0.001) (Table 3).

A large proportion of male and female adult participants were willing to perform ventilation on close family relatives [mother (male = 97 %, female = 100 %), father (male = 97.6 %, female = 98.7 %), brother (male = 100 %, female = 97.9 %), sister (male = 95.6 %, female = 100 %)]. Unlike adolescent participants, adults did not differ significantly between male and females in willingness to ventilate a grandmother or grandfather (P = 0.38 and 0.97 respectively). Adults also had higher willingness response rates for all victims (Table 4).

**Table 4:** Willingness of adults to perform ventilations on hypothesized victims by sex

Victim identity	Male Respondent		Female Respondent		Chi^2^ P (* = Fishers exact)	Odds Ratio	95 % CI
						Male/Female (* = Female/Male)	
	N	%	N	%			
Mother	98	97.0	91	100.0	*0.25	*1.03	1.00 – 1.06
Father	81	97.6	77	98.7	*1	*1.89	0.17 – 21.28
Brother	134	100.0	95	97.9	*0.18	1.02	0.99 – 1.05
Sister	129	95.6	100	100.0	*0.05	*1.04	1.01 – 1.09
Grandmother	39	90.7	32	97.0	*0.38	*3.23	2.86 – 33.33
Grandfather	29	85.3	18	85.7	*0.97	1.03	0.22 – 4.76
Aunt	103	84.4	90	94.7	0.02	*3.33	1.19 – 9.09
Uncle	118	95.9	54	56.3	<0.001	18.36	6.88 – 48.98
Male friend	124	93.2	36	36.0	<0.001	24.49	11.11 – 53.99
Female friend	84	68.9	92	93.9	<0.001	*7.14	2.78 – 16.67
Stranger**	118	87.4	31	31.0	<0.001	15.45**	7.97 – 29.95

**Sex of stranger not stated

## Discussion

This study was part of a broader set of feasibility research to establish a village-based first response system including drowning in rural Bangladesh. A previous study showed it was possible to train selected participants to deliver CPR that included mouth-to-mouth ventilation and first response skills in the low resource setting of rural Bangladesh [8]. This study examined the willingess of the participants to provide mouth-to-mouth ventilation to various related and non-related recipients in hypothesized CPR scenarios. In this study of 721 adolescent and adult participants there were large differences in the willingness to perform mouth-to-mouth ventilation on hypothetical victims. The scenarios included immediate family members, extended family members, friends and strangers. Participants were more willing to administer ventilation to a hypothetical victim whose sex was the same as their own, as well as when the hypothetical victim was a close family member. The lack of willingness was greatest when the hypothetical victim was a friend of the opposite sex or a stranger. The differences were greater in all victim scenarios in adolescent participants compared with adults.

We are unaware of studies conducted on the provision of CPR which include mouth-to-mouth ventilation in low income countries outside of hospital or other clinical settings. Therefore, little is known about the feasibility of introducing a large scale community-based CPR response program for drowning and the potential for success in such countries. Previous studies have suggested there may be cultural barriers limiting willingness to perform mouth-to-mouth ventilation in countries with Islam as the predominant religion. This study confirms a potential barrier to willingness in Bangladesh, another Islamic-majority country, when the participant is presented with a variety of hypothetical scenarios. It also shows a high proportion of participants would be willing to perform mouth-to-mouth ventilation on family members, and friends if they received the appropriate training.

The sex of the scenario victim significantly influenced willingness to administer ventilation in nearly all scenarios. In rural Bangladesh conservative attitudes limit physical interaction between sexes after puberty and before marriage. Even in the selected sample of youthful rural participants with primary school education, differences due to sex are apparent even with close family members. However, the proportion of participants willing to administer ventilations on family members of the opposite sex was high (>79 % willingness to ventilate mother, father, sister, or brother).

Beyond the immediate family, differences were greater. Adolescents had a large difference between the willingness of a boy and girl to administer mouth-to-mouth ventilation on their grandfather (80 % vs 47 % respectively) and grandmother (63 % vs 81 % respectively). In Bangladesh (as in many other Asian countries) notions of respect for older people or those in higher authority – even among close family members – often limits physical interaction, potentially negatively influencing a young person’s willingness to perform ventilation.

Willingness varied for male and female participants in both adolescent and adult age groups to perform ventilation on an aunt or an uncle of the opposite gender. This may be due to use of the words aunt and uncle in Bangladesh. Family friends and distant relatives of the same age as parents are often referred to as aunt or uncle, potentially creating ambiguity surrounding the relationship of the victim to the rescuer. Both adolescent and adult participants were less willing to ventilate a friend of the opposite sex.

There was no difference in the adults who were willing to perform mouth-to-mouth ventilation in at least 85 % of all circumstances. Adults expressed more willingness to ventilate a broader range of drowning victims than adolescents. However in rural Bangladesh, adolescents are most likely to be in close proximity to a drowning in progress. A study in the same rural research site showed a high rate of water rescues conducted by adolesents [10]. Given the need for adolescents to be the primary first responders for drowning, further research will be needed to examine ways willingness to deliver mouth-to-mouth ventilation can be increased for them.

There may be greater willingness to perform compression-only CPR as it does not involve mouth-to-mouth contact. Participants were encouraged to do compression only CPR if they felt uncomfortable giving mouth-to mouth ventilations. However, the previous research showed the great majority of response events in Bangladesh that require CPR are hypoxic events due to drowning [1]. Guidelines from both the European Resuscitation Council (ERC) and American Heart Foundation (AHF) state that mouth-to-mouth ventilation is required for resuscitation of drowning victims [4]. The AHF notes that compression-only CPR is likely to be less effective and should be avoided [11].

Limitations to the study include the non-representative nature of the participants, and the relatively small sample size. Trainees were selected for a willingness to learn first response, potentially selecting for those with a greater willingness to perform mouth-to-mouth ventilation as part of the first response training. A further limitation is the data describes a professed willingness in scenarios with hypothesized victims rather than real response events. The willingness to perform mouth-to-mouth ventilation seen in this study may not be the same broadly for adolescents and adults in the community. Finally, the responses given in this study were part of a pre-test before beginning the training. It is unknown if participation in the training had an effect on willingness following receipt of training. This is currently under investigation.

## Conclusions

The study found that a large proportion of selected participants were willing to ventilate a close family member or friend. However, these participants were selected on the basis of education, higher social status and youthful age and do not reflect the average rural population in Bangladesh. Despite the unrepresentative nature of the participants, the proportion of participants willing to give ventilation is comparable to a number of studies conducted in high-income countries. A follow-up study is underway to determine whether pre-course willingness examined in this study has been increased as a result of achieving competence in CPR. These results will provide further information on feasibility of a first responder programme in rural Bangladesh. Research is ongoing as well to evaluate clinical outcomes and provide evidence whether graduates reduce morbidity and mortality through effective first response.
